# Eosinophilic granuloma of the clavicle in an 11-year-old Chinese girl: A case report

**DOI:** 10.1097/MD.0000000000034139

**Published:** 2023-06-30

**Authors:** Qian-Dui Chen, Shuang-Zhu Lin, Jie Zhou, Xiao-Xiao Chen, Rui-Tong Yang, Xinyao Wang, Jia-Yi Li, Xiao-Chun Feng, Yu-Shu Liu, Kai Jiang, Jin-Hua Feng

**Affiliations:** a College of Integrated Chinese and Western Medicine, Changchun University of Chinese Medicine, Changchun, Jilin Province, China; b Diagnosis and Treatment Center for Children, The Affiliated Hospital of Changchun University of Chinese Medicine, Changchun, Jilin Province, China; c Diagnosis and Treatment Center for Heart, The Affiliated Hospital of Changchun University of Chinese Medicine, Changchun, Jilin Province, China; d Pediatrics of Traditional Chinese Medicine, College of Traditional Chinese Medicine, Changchun University of Chinese Medicine, Changchun, Jilin Province, China; e The Third Affiliated Hospital of Changchun University of Chinese Medicine, Changchun, Jilin Province, China; f The Affiliated Hospital of Changchun University of Chinese Medicine, Changchun, Jilin Province, China.

**Keywords:** case report, clavicle, diagnosis, eosinophilic granuloma (EG)

## Abstract

Eosinophilic granuloma (EG) – the most common form of Langerhans cell histiocytosis – occurs rarely, and manifestations with only rib and clavicle involvement are extremely rare. EG symptoms often include pain, swelling, and soft tissue mass. The clinical diagnosis of bone EG is complex, and the differential diagnosis includes Ewing sarcoma, tuberculosis, multiple myeloma, lymphoma, primary bone malignancy, and other osteolytic lesions.

**Patient’s concern:**

The patient was an 11-year-old female who found a subcutaneous mass at the junction of the right clavicle and sternum 2 days before presenting at the clinic without apparent triggers. Initially, we considered a subcutaneous cyst or inflammatory mass. Color ultrasound and computed tomography examination revealed osteomyelitis. Finally, the patient was diagnosed with EG after a pathological tissue biopsy, and the child recovered after surgery and anti-infective treatment.

**Diagnosis:**

The patient underwent surgery to remove the tumor at a specialist hospital and was diagnosed with EG by pathological examination.

**Intervention:**

The patient went to a specialist hospital for surgery to remove the mass and underwent anti-infective treatment.

**Outcomes:**

The patient recovered after surgical resection and antibiotic treatment.

**Lessons:**

In this report, we emphasize that the clinical presentation of EG in children is not specific. Furthermore, examining age, history, presence of symptoms, and the number of sites is essential to make a correct diagnosis, and a histological examination is necessary to confirm the diagnosis.

## 1. Introduction

Eosinophilic granulomas (EG), Letterer–Siwe disease, and Hand-Schuller-Christian disease are collectively known as Langerhans cell tissue histiocytosis (LCH) and are rare childhood diseases characterized by abnormal proliferation of Langerhans cells that can affect multiple organs, such as the skin, bones, liver, or lungs.^[1,2]^ The annual incidence in the pediatric age group is 3 to 5/1000,000 cases.^[3]^ EG is the most common form of LCH, occurring very rarely and accounting for <1% of all bone tumor-like lesions, often affecting single or multiple bones, most commonly the skull, mandible, ribs, pelvis, or long bones.^[1]^

EG is the mildest form of localized LCH, and there are 2 common clinical forms: a single bone lesion (solitary) or multiple bone lesions (multiple).^[4]^ EG mainly affects children, with the highest frequency in patients aged 5 to 10 years, and is most prevalent in males.^[5,6]^ Solitary EG comprises 70% of cases,^[7]^ with the most common site being the mandible (30%), followed by the skull (21%), vertebrae (13%), and extremities (13%), and rib and clavicle involvement are extremely rare (6%).^[4]^ The clinical course of bone EG is variable, and in most patients, EG is a self-limiting process with alternating stages of relapse-remission and spontaneous resolution. Patients with bone involvement often present with pain, swelling, and soft tissue masses, and localized bone pain is the initial symptom in 50 to 90% of patients with bone lesions.^[8]^

## 2. Case presentation

An 11-year-old female presented with a subcutaneous mass in the right clavicle without any obvious triggers. Discovered 2 days prior, visited our hospital on January 20, 2023. The mass was approximately 3 cm × 3 cm and located at the junction of the right collarbone and sternum; the skin color and temperature were normal. The mass was raised, and the area was mildly tender. There was no noticeable fluctuation, rash, desquamation, or fistula. The child reported symptoms of general malaise and had no symptoms such as fever, trauma, vomiting, diarrhea, and coma since the onset of the illness.

### 2.1. Physical examination

The child’s skin and mucous membranes were normal. The skin was not edematous, and the superficial lymph nodes were not palpated nor enlarged. The pharynx was not congested, and the tonsils were not enlarged. The patient’s trachea was centered, and the thoracic cage was regular. The bilateral thyroid gland was not palpated nor enlarged. A mass with smooth edges, measuring approximately 3 cm × 3 cm, was observed on the right chest. The patient’s skin temperature was normal, with mild tenderness. There were no apparent adhesions with surrounding tissues. The patient’s breathing and intercostal space were normal, with clear breath sounds in both lungs and no dry or wet rale. The apical beat and cardiac dullness were normal. The patient had a heart rate of about 100 beats/min, an aligned rhythm, no subxiphoid pulsation, and no pathological murmur. The liver, spleen, and gallbladder were not palpable, and the spine was normal. The patient’s physical activity was regular, and the physiological reflexes were present and pathological reflexes were not elicited.

### 2.2. Laboratory and imaging results

Routine blood analysis, performed on January 20, 2023, revealed: WBC: 8.69 × 10^9^/L, NEUT%: 70.40%, LYMPH%: 21.90%, MONO%: 7.50%, RBC: 4.25 × 10^12^/L, PLT: 295 × 10^9^/L, and hs-CRP: 18.40 mg/L. The results for myocardial enzymes, liver function, renal function, tuberculosis antibodies, and procalcitonin were all normal.

### 2.3. Imaging examination

**Figure 1.**
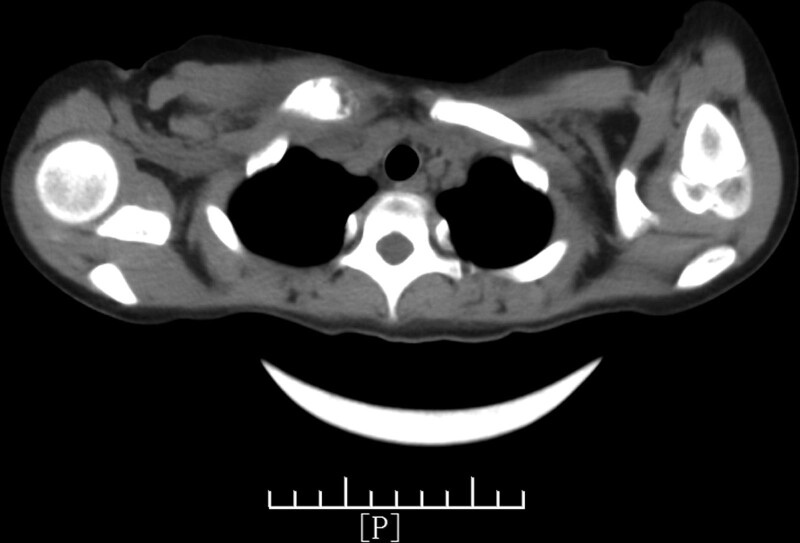
Computed tomography scan of the sternum. The right clavicle sternal end is thicker than the strategy. The density is uneven with clear osteolytic changes and the cortical morphology is disordered. The surrounding soft tissue is thickened and the local density of the upper edge of the sternum is uneven.

**Figure 2.**
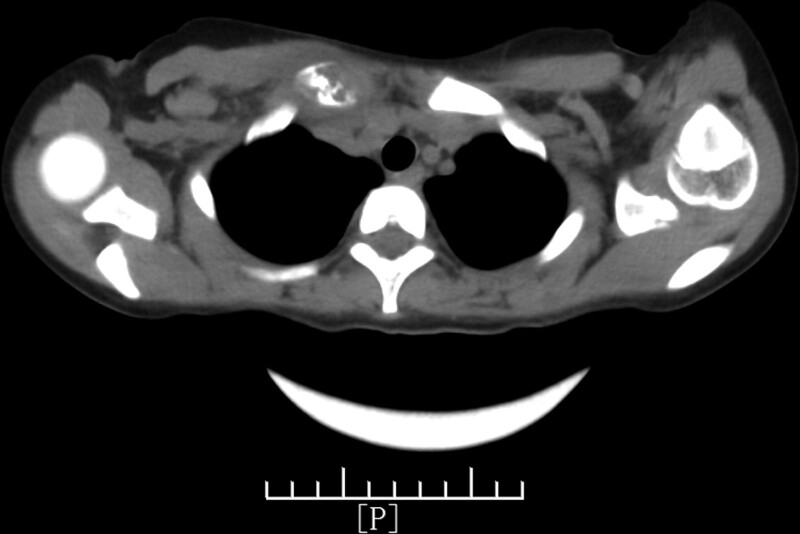
Computed tomography scan of the sternum. The right clavicle sternal end is thicker than the strategy. The density is uneven with clear osteolytic changes and the cortical morphology is disordered. The surrounding soft tissue is thickened and the local density of the upper edge of the sternum is uneven.

**Figure 3.**
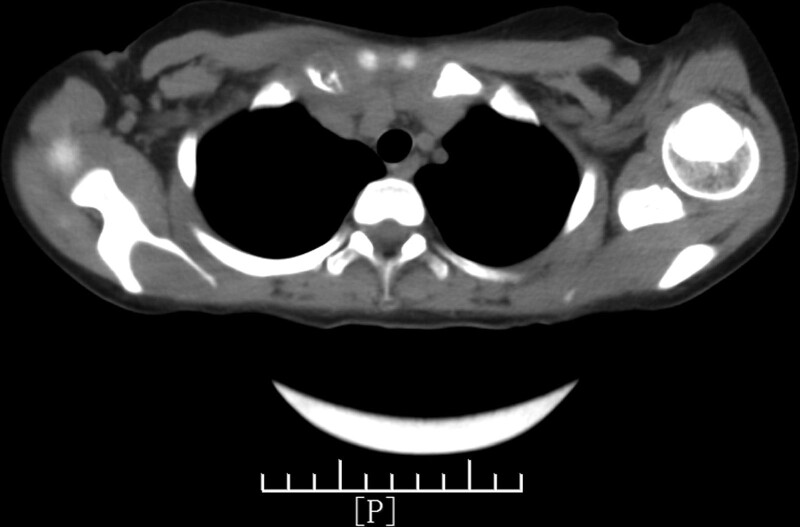
Computed tomography scan of the sternum. The right clavicle sternal end is thicker than the strategy. The density is uneven with clear osteolytic changes and the cortical morphology is disordered. The surrounding soft tissue is thickened and the local density of the upper edge of the sternum is uneven.

The superficial color ultrasound showed a heterogeneous echo near the sternoclavicular joint of the right clavicle with a size of approximately 2.1 cm × 1.7 cm, with a rich internal blood flow signal. The CT of the sternum revealed that the right clavicle sternum end was thicker than the contralateral side, the density was uneven, osteolytic changes were observed, the cortical morphology was poor, the surrounding soft tissue was thickened, the local density of the upper edge of the sternum was uneven, and the morphological densities of the remaining sternum and left clavicle were not abnormal (Figs. 1–3).

The patient was injected with amoxicillin sodium and clavulanate potassium at 1.2 g thrice on the day of admission as an anti-infection treatment. One day after treatment, her general malaise symptoms were significantly relieved; however, we recommended that the child goes to a specialist hospital for further treatment. Through follow-up, we learned that she underwent a biopsy and surgical removal of the mass in the specialist hospital. The pathological tissue results showed hyperplasia of inflammatory granulation tissue, multifocal microabscess formation, and local granuloma formation. Immunohistochemistry of the masses showed Ki-67 (+10%), CD1a(+), Langerin(+), CD68 (scattered +), and S-100(+). After surgery, the patient underwent aggressive anti-infective treatment and was eventually discharged from the hospital.

## 3. Discussion

EG was initially described by Otani and Ehrhch,^[9]^ followed by Lichtenstein and Jaffe.^[7]^ The pathogenesis of EG has been controversial, with some authors suggesting that EG is an immune system disorder that manifests with hypersensitivity to unknown antigens and stimulation of the histiocyte-macrophage system.^[10,11]^ The clonal proliferation of Langerhans cells and the increased expression of several tumor-associated factors have resulted in speculations that EG is a tumor. In addition, although the specific pathogenic microorganism has not been identified due to its self-limitation and response to steroids and antibiotics, some researchers suspect the disease’s origin is an inflammatory response.^[8]^

Clinically, the diagnosis of skeletal EG is difficult. EG is considered a great imitator, and from the radiological point of view, its performance could be more specific. In radiology, skeletal EG manifests as lytic with destructive periosteum lesions, which may be poorly demarcated with irregular margins.^[12]^ Differential diagnosis for EG includes Ewing sarcoma, tuberculosis, multiple myeloma, lymphoma, primary bone malignancy and other osteolytic lesions. Diagnosis is often unclear by clinical symptoms or imaging examination alone. Histological sampling should be performed after adequate local imaging, an essential step in diagnosing and treating bone lesions. Furthermore, the trauma of biopsy can lead to local symptoms relief of such diseases.^[13]^ The pathological tissue of EG can be observed under the microscope in addition to pathological Langerhans cells and inflammatory cells such as lymphocytes, eosinophils, and macrophages. Immunohistochemical positivity for CD1a, S100, and Langerin has been observed in biopsy or surgical excision tissues.^[4]^

We initially considered a local soft tissue cyst as no other symptoms existed. The elevated neutrophils and C-reactive protein levels indicated bacterial infection, and the local color ultrasound results made us further consider the possibility of osteomyelitis. Furthermore, sternal CT showed thickened sternal ends of the right clavicle with osteolytic changes and thickening in surrounding soft tissues. Considering the older age and female sex of the patient, these findings supported the diagnosis of osteomyelitis. At the same time, compared with the typical case of EG, our child had local swelling, pain, and soft tissue masses, and the clinical symptoms was consistent with the typical bone EG. However, the child was beyond the age of onset of the disease, and the onset site was in the collarbone, which was rarer clinically. Without the support of pathological tissue biopsy results, we could not confirm the diagnosis based on imaging and ultrasound alone. Ultimately, we confirmed the bone EG based on the immunohistochemical results.

There is no standard treatment regimen for skeletal EG; treatment options may vary depending on the disease location and extent. In mono-focal bone EG, close observation may be preferred, but surgical resection, radiation therapy, and intralesional steroid therapy are also options. Patients with the mono-focal bone disease have a reasonably good prognosis, and systemic chemotherapy is recommended for patients with multiple system involvement.^[14]^ Our child finally received surgical treatment, and actively fought against the infection after the operation, and finally recovered.

## 4. Conclusion

In conclusion, we present a rare case of skeletal EG of the clavicle in an 11-year-old child. This case suggests that the clinical presentation in such children needs to be more specific. Age, history, presence or absence of symptoms, and the number of sites are essential in diagnosing correctly. However, a definitive diagnosis can only be made through histological examination.

## Acknowledgments

We thank the patient and their family for their participation in this study. There is no conflict of interest for all authors.

## Author contributions

**Conceptualization:** Xiaochun Feng, Yushu Liu, Kai Jiang.

**Project administration:** Xiaochun Feng, Yushu Liu, Jinhua Feng.

**Resources:** Shuangzhu Lin, Jie Zhou, Xiaoxiao Chen, Kai Jiang.

**Validation:** Xinyao Wang.

**Writing – original draft:** Qiandui Chen, Jie Zhou, Xiaoxiao Chen, Ruitong Yang, Jia-Yi Li.
